# 4-(Cyano­meth­yl)anilinium chloride

**DOI:** 10.1107/S1600536810018076

**Published:** 2010-05-22

**Authors:** Jin-rui Lin

**Affiliations:** a Ordered Matter Science Research Center, Southeast University, Nanjing 210096, People’s Republic of China

## Abstract

The crystal structure of the title compound, C_8_H_9_N_2_^+^·Cl^−^, is stabilized by N—H⋯Cl hydrogen bonds.

## Related literature

For background to phase transition materials, see: Li *et al.* (2008 ▶); Zhang *et al.* (2009 ▶).
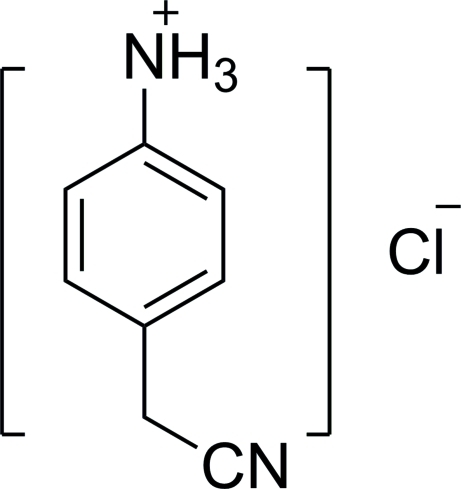


## Experimental

### 

#### Crystal data

C_8_H_9_N_2_^+^·Cl^−^
*M* *_r_* = 168.62
Monoclinic, 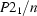

*a* = 5.4348 (12) Å
*b* = 8.5630 (18) Å
*c* = 18.000 (4) Å
β = 93.734 (16)°
*V* = 835.9 (3) Å^3^
*Z* = 4
Mo *K*α radiation
μ = 0.39 mm^−1^
*T* = 293 K
0.45 × 0.28 × 0.25 mm

#### Data collection

Rigaku SCXmini diffractometer
Absorption correction: multi-scan (*CrystalClear*; Rigaku, 2005 ▶) *T*_min_ = 0.5, *T*_max_ = 0.5
8241 measured reflections
1890 independent reflections
1593 reflections with *I* > 2σ(*I*)
*R*_int_ = 0.036

#### Refinement

*R*[*F* ^2^ > 2σ(*F* ^2^)] = 0.039
*wR*(*F* ^2^) = 0.139
*S* = 1.18
1890 reflections
101 parameters
H-atom parameters constrained
Δρ_max_ = 0.50 e Å^−3^
Δρ_min_ = −0.54 e Å^−3^

### 

Data collection: *CrystalClear* (Rigaku, 2005 ▶); cell refinement: *CrystalClear*; data reduction: *CrystalClear*; program(s) used to solve structure: *SHELXS97* (Sheldrick, 2008 ▶); program(s) used to refine structure: *SHELXL97* (Sheldrick, 2008 ▶); molecular graphics: *SHELXTL* (Sheldrick, 2008 ▶); software used to prepare material for publication: *PRPKAPPA* (Ferguson, 1999 ▶).

## Supplementary Material

Crystal structure: contains datablocks I, global. DOI: 10.1107/S1600536810018076/jh2157sup1.cif

Structure factors: contains datablocks I. DOI: 10.1107/S1600536810018076/jh2157Isup2.hkl

Additional supplementary materials:  crystallographic information; 3D view; checkCIF report

## Figures and Tables

**Table 1:** Hydrogen-bond geometry (Å, °)

*D*—H⋯*A*	*D*—H	H⋯*A*	*D*⋯*A*	*D*—H⋯*A*
N1—H1*B*⋯Cl1	0.89	2.31	3.1638 (17)	162
N1—H1*A*⋯Cl1^i^	0.89	2.32	3.2061 (16)	177
N1—H1*C*⋯Cl1^ii^	0.89	2.29	3.1700 (17)	168

Symmetry codes: (i) 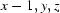; (ii) 
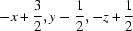
.

**Fig. 1.**
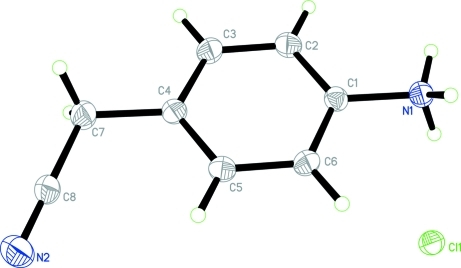
The molecular structure of the title compound, showing the atomic numbering scheme. Displacement ellipsoids are drawn at the 30% probability level.

**Fig. 2.**
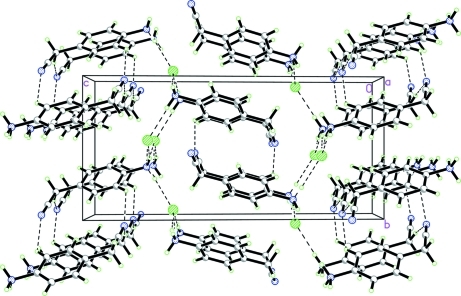
A view of the packing of the title compound, stacking along the a axis. Dashed lines indicate hydrogen bonds.

## supplementary crystallographic information

### Comment

Most non-hydrogen atoms of the 4-(cyanomethyl)anilinium were coplanar, with the mean deviation from plane of 0.0320 and N_2_—C_8_—C_7_—C_4_ torsion angle of 114 (37)°. The strong π-π packing interactions of benzene rings with Cg(1)···Cg(1) of 3.487 Å (Cg(1) is the centroid of benzene ring) stabilized the crystal structure. The N—H···Cl hydrogen bonding with the N···Cl distances from 3.1638 (17) Å to 3.2061 (17) Å link the molecules into infinite two-dimensional plane.

As a continuation of our study of phase transition materials, including organic ligands (Li *et al.*, 2008), metal-organic coordination compounds (Zhang *et al.*, 2009 ),the dielectric constant of 4-(cyanomethyl)anilinium chloride compound as a function of temperature indicates that the permittivity is basically temperature-independent (dielectric constant equaling to 5.3 to 21.1), suggesting that this compound should be not a real ferroelectrics or there may be no distinct phase transition occurred within the measured temperature range.

### Experimental

Single crystals (average size: 0.7×0.8×1.0 mm) of 4-(cyanomethyl)anilinium chloride were prepared by slowevaporation at room temperature of an ethanol solution of equal molar for 4 days.

### Refinement

Positional parameters of all the H atoms were calculated geometrically and were allowed to ride on the C and N atoms to which they are bonded, with *U*_iso_(H) = 1.2U_eq_(C),*U*_iso_(H) = 1.5U_eq_(N).

### Crystal data

**Table:**

C_8_H_9_N_2_^+^·Cl^−^	*F*(000) = 352
*M**_r_* = 168.62	*D*_x_ = 1.340 Mg m^−^^3^
Monoclinic, *P*2_1_/*n*	Mo *K*α radiation, λ = 0.71073 Å
Hall symbol: -P 2yn	Cell parameters from 2330 reflections
*a* = 5.4348 (12) Å	θ = 3.2–27.6°
*b* = 8.5630 (18) Å	µ = 0.39 mm^−^^1^
*c* = 18.000 (4) Å	*T* = 293 K
β = 93.734 (16)°	Prism, orange
*V* = 835.9 (3) Å^3^	0.45 × 0.28 × 0.25 mm
*Z* = 4	

### Data collection

**Table:**

Rigaku SCXmini diffractometer	1890 independent reflections
Radiation source: fine-focus sealed tube	1593 reflections with *I* > 2σ(*I*)
graphite	*R*_int_ = 0.036
Detector resolution: 13.6612 pixels mm^-1^	θ_max_ = 27.5°, θ_min_ = 2.3°
CCD_Profile_fitting scans	*h* = −7→7
Absorption correction: multi-scan (*CrystalClear*; Rigaku, 2005)	*k* = −11→11
*T*_min_ = 0.5, *T*_max_ = 0.5	*l* = −23→23
8241 measured reflections	

### Refinement

**Table:**

Refinement on *F*^2^	Primary atom site location: structure-invariant direct methods
Least-squares matrix: full	Secondary atom site location: difference Fourier map
*R*[*F*^2^ > 2σ(*F*^2^)] = 0.039	Hydrogen site location: inferred from neighbouring sites
*wR*(*F*^2^) = 0.139	H-atom parameters constrained
*S* = 1.18	*w* = 1/[σ^2^(*F*_o_^2^) + (0.0842*P*)^2^] where *P* = (*F*_o_^2^ + 2*F*_c_^2^)/3
1890 reflections	(Δ/σ)_max_ < 0.001
101 parameters	Δρ_max_ = 0.50 e Å^−^^3^
0 restraints	Δρ_min_ = −0.54 e Å^−^^3^

### Special details

**Table:**

Geometry. All esds (except the esd in the dihedral angle between two l.s. planes) are estimated using the full covariance matrix. The cell esds are taken into account individually in the estimation of esds in distances, angles and torsion angles; correlations between esds in cell parameters are only used when they are defined by crystal symmetry. An approximate (isotropic) treatment of cell esds is used for estimating esds involving l.s. planes.
Refinement. Refinement of *F*^2^ against ALL reflections. The weighted *R*-factor wR and goodness of fit *S* are based on *F*^2^, conventional *R*-factors *R* are based on *F*, with *F* set to zero for negative *F*^2^. The threshold expression of *F*^2^ > σ(*F*^2^) is used only for calculating *R*-factors(gt) etc. and is not relevant to the choice of reflections for refinement. *R*-factors based on *F*^2^ are statistically about twice as large as those based on *F*, and *R*- factors based on ALL data will be even larger.

### Fractional atomic coordinates and isotropic or equivalent isotropic displacement parameters (Å^2^)

**Table:**

	*x*	*y*	*z*	*U*_iso_*/*U*_eq_	
C1	0.4618 (3)	0.31846 (19)	0.11664 (9)	0.0300 (4)	
C2	0.2846 (3)	0.3656 (2)	0.06362 (11)	0.0392 (4)	
H2	0.1527	0.4266	0.0768	0.047*	
C3	0.3045 (3)	0.3213 (2)	−0.00980 (11)	0.0399 (5)	
H3	0.1846	0.3524	−0.0460	0.048*	
C4	0.5017 (3)	0.2308 (2)	−0.02981 (9)	0.0318 (4)	
C5	0.6768 (3)	0.1847 (2)	0.02470 (10)	0.0381 (4)	
H5	0.8087	0.1233	0.0119	0.046*	
C6	0.6587 (3)	0.2288 (2)	0.09839 (10)	0.0375 (4)	
H6	0.7780	0.1981	0.1348	0.045*	
C7	0.5134 (4)	0.1847 (3)	−0.11122 (11)	0.0424 (5)	
H7A	0.3717	0.1201	−0.1257	0.051*	
H7B	0.5031	0.2784	−0.1416	0.051*	
C8	0.7369 (4)	0.1002 (2)	−0.12648 (10)	0.0380 (4)	
N1	0.4471 (3)	0.36295 (19)	0.19498 (8)	0.0339 (4)	
H1A	0.3056	0.4126	0.2005	0.051*	
H1B	0.5727	0.4256	0.2087	0.051*	
H1C	0.4541	0.2776	0.2233	0.051*	
N2	0.9120 (4)	0.0349 (3)	−0.13867 (12)	0.0593 (6)	
Cl1	0.94611 (8)	0.55382 (5)	0.21151 (3)	0.0395 (2)	

### Atomic displacement parameters (Å^2^)

**Table:**

	*U*^11^	*U*^22^	*U*^33^	*U*^12^	*U*^13^	*U*^23^
C1	0.0310 (9)	0.0303 (8)	0.0290 (8)	−0.0016 (7)	0.0037 (7)	0.0014 (7)
C2	0.0324 (9)	0.0462 (11)	0.0391 (10)	0.0129 (8)	0.0023 (7)	−0.0008 (8)
C3	0.0344 (10)	0.0505 (11)	0.0339 (10)	0.0123 (8)	−0.0045 (7)	0.0023 (8)
C4	0.0309 (9)	0.0335 (9)	0.0310 (9)	0.0017 (7)	0.0011 (7)	0.0010 (7)
C5	0.0318 (9)	0.0460 (11)	0.0362 (10)	0.0116 (8)	0.0005 (7)	−0.0027 (8)
C6	0.0324 (9)	0.0471 (10)	0.0322 (9)	0.0095 (8)	−0.0030 (7)	0.0022 (8)
C7	0.0409 (11)	0.0528 (12)	0.0327 (10)	0.0119 (9)	−0.0024 (8)	−0.0024 (8)
C8	0.0395 (11)	0.0464 (10)	0.0279 (9)	0.0031 (9)	0.0011 (7)	0.0002 (8)
N1	0.0347 (8)	0.0367 (8)	0.0305 (8)	0.0031 (6)	0.0034 (6)	0.0007 (6)
N2	0.0484 (12)	0.0836 (15)	0.0463 (11)	0.0210 (10)	0.0059 (9)	−0.0014 (10)
Cl1	0.0360 (3)	0.0418 (3)	0.0404 (3)	0.00352 (17)	−0.0006 (2)	−0.00759 (18)

### Geometric parameters (Å, °)

**Table:**

C1—C2	1.372 (3)	C5—H5	0.9300
C1—C6	1.374 (2)	C6—H6	0.9300
C1—N1	1.468 (2)	C7—C8	1.455 (3)
C2—C3	1.386 (3)	C7—H7A	0.9700
C2—H2	0.9300	C7—H7B	0.9700
C3—C4	1.389 (2)	C8—N2	1.137 (3)
C3—H3	0.9300	N1—H1A	0.8900
C4—C5	1.380 (3)	N1—H1B	0.8900
C4—C7	1.523 (3)	N1—H1C	0.8900
C5—C6	1.389 (3)		
			
C2—C1—C6	121.37 (16)	C1—C6—H6	120.5
C2—C1—N1	120.80 (16)	C5—C6—H6	120.5
C6—C1—N1	117.82 (16)	C8—C7—C4	113.47 (16)
C1—C2—C3	119.20 (16)	C8—C7—H7A	108.9
C1—C2—H2	120.4	C4—C7—H7A	108.9
C3—C2—H2	120.4	C8—C7—H7B	108.9
C2—C3—C4	120.63 (17)	C4—C7—H7B	108.9
C2—C3—H3	119.7	H7A—C7—H7B	107.7
C4—C3—H3	119.7	N2—C8—C7	179.6 (3)
C5—C4—C3	118.92 (16)	C1—N1—H1A	109.5
C5—C4—C7	122.68 (16)	C1—N1—H1B	109.5
C3—C4—C7	118.39 (16)	H1A—N1—H1B	109.5
C4—C5—C6	120.85 (17)	C1—N1—H1C	109.5
C4—C5—H5	119.6	H1A—N1—H1C	109.5
C6—C5—H5	119.6	H1B—N1—H1C	109.5
C1—C6—C5	119.02 (17)		
			
C6—C1—C2—C3	0.2 (3)	C2—C1—C6—C5	−0.3 (3)
N1—C1—C2—C3	−179.95 (17)	N1—C1—C6—C5	179.84 (17)
C1—C2—C3—C4	−0.3 (3)	C4—C5—C6—C1	0.5 (3)
C2—C3—C4—C5	0.5 (3)	C5—C4—C7—C8	−5.2 (3)
C2—C3—C4—C7	179.66 (19)	C3—C4—C7—C8	175.67 (19)
C3—C4—C5—C6	−0.6 (3)	C4—C7—C8—N2	−114 (37)
C7—C4—C5—C6	−179.74 (18)		

### Hydrogen-bond geometry (Å, °)

**Table:**

*D*—H···*A*	*D*—H	H···*A*	*D*···*A*	*D*—H···*A*
N1—H1B···Cl1	0.89	2.31	3.1638 (17)	162
N1—H1A···Cl1^i^	0.89	2.32	3.2061 (16)	177
N1—H1C···Cl1^ii^	0.89	2.29	3.1700 (17)	168

Symmetry codes: (i) *x*−1, *y*, *z*; (ii) −*x*+3/2, *y*−1/2, −*z*+1/2.
